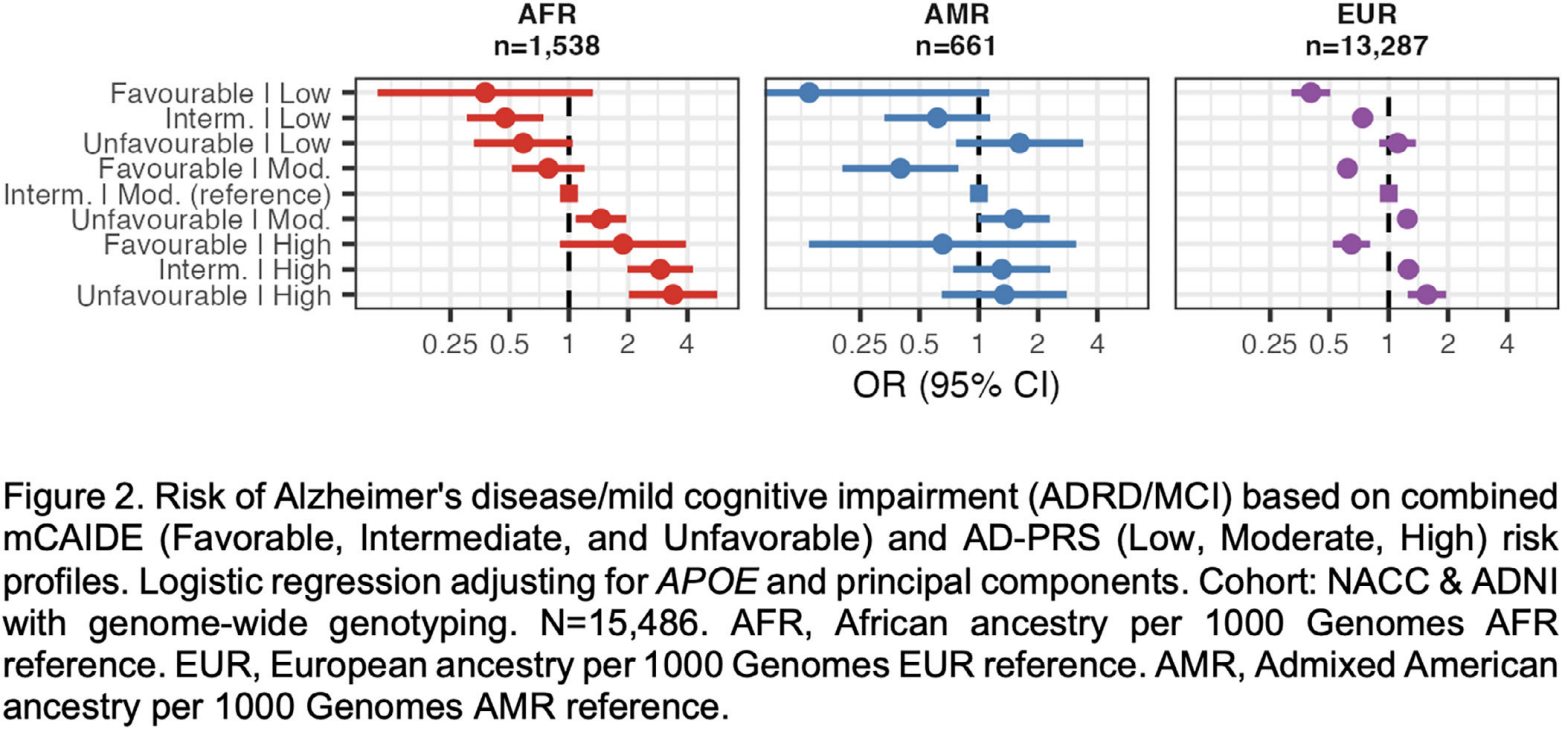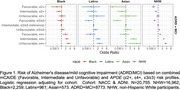# Modifiable risk factors mitigate genetic risk for AD across diverse populations

**DOI:** 10.1002/alz70855_107321

**Published:** 2025-12-24

**Authors:** Paulina Tolosa Tort, Meri Okorie, Ana I Boeriu, Caroline Jonson, Jennifer S. Yokoyama, Kristine Yaffe, Shea J Andrews

**Affiliations:** ^1^ Department of Psychiatry and Behavioral Sciences, University of California ‐ San Francisco, San Francisco, CA, USA; ^2^ University of California, San Francisco, San Francisco, CA, USA; ^3^ Global Brain Health Institute, University of California, San Francisco, San Francisco, CA, USA; ^4^ Memory and Aging Center, Weill Institute for Neurosciences, University of California San Francisco, San Francisco, CA, USA; ^5^ Department of Psychiatry, University of California San Francisco, San Francisco, CA, USA; ^6^ Department of Neurology, University of California, San Francisco, San Francisco, CA, USA; ^7^ University of California, San Francisco, Weill Institute for Neurosciences, San Francisco, CA, USA; ^8^ Department of Epidemiology, University of California, San Francisco, San Francisco, CA, USA

## Abstract

**Background:**

Alzheimer's disease (AD) is influenced by both genetic and environmental factors. Notably, genetic risk may be moderated by modifiable risk factors. The APOE*ε4 allele is the strongest genetic risk factor, while polygenic risk scores (PRS) capture additional genetic liability. Dementia clinical risk scores (CRS) integrate clinical and lifestyle risk factors for risk stratification. However, as PRS and CRS demonstrate population‐specific effects, the extent to which modifiable risk factors moderate genetic risk in diverse populations needs to be determined. Here, we investigate whether modifiable risk profiles moderate the effects of *APOE* and AD‐PRS on AD and mild cognitive impairment (MCI) across diverse populations.

**Method:**

Using data from 20,755 participants in NACC and ADNI (aged 73 ± 8 years; 56% female; 82% non‐Hispanic White (NHW), 11% Black, 4.6% Latinx, 2.8% Asian), we constructed the modified Cardiovascular Risk Factors, Aging, and Incidence of Dementia (mCAIDE) risk score, incorporating age, sex, education, obesity, hypertension, and hypercholesterolemia. In a subset of participants with genome‐wide genotyping (*N* = 15,486), we generated a cross‐ancestry AD‐PRS using PRS‐CSx (excluding *APOE*). Both mCAIDE and AD‐PRS were categorized into tertiles (favorable/intermediate/unfavorable and low/moderate/high). mCAIDE and *APOE* (ε2+, ε4+, ε3/ε3) were combined into nine risk categories, with intermediate‐ε3/ε3 as the reference. A similar approach was used for mCAIDE and AD‐PRS. Additive interactions were assessed using multivariate logistic regression, stratified by self‐reported race/ethnicity for the *APOE* analysis and estimated genetic ancestry for the AD‐PRS.

**Result:**

A favorable mCAIDE profile lowered APOE*ε4‐associated risk, while an unfavorable profile reduced APOE*ε2's protective effect, particularly in NHW and Black self‐reported race (Figure 1). Similarly, a favorable mCAIDE profile mitigated the risk from high AD‐PRS, whereas an unfavorable profile weakened the protection of low AD‐PRS (Figure 2). These trends were most evident in 1KG‐EUR‐like and 1KG‐AFR‐like ancestry groups. The moderating effect of modifiable risk factors on genetic risk was less consistent in other populations, likely due to smaller sample sizes.

**Conclusion:**

Our findings show that modifiable risk factors moderate genetic risk for AD across diverse populations, highlighting the need to integrate genetic and environmental factors into personalized risk assessments.